# Nectandrin B-mediated activation of the AMPK pathway prevents cellular senescence in human diploid fibroblasts by reducing intracellular ROS levels

**DOI:** 10.18632/aging.102013

**Published:** 2019-06-14

**Authors:** Hyun-Jin Jang, Kyeong Eun Yang, Won Keun Oh, Song-I Lee, In-Hu Hwang, Kyung-Tae Ban, Hwa-Seung Yoo, Jong-Soon Choi, Eui-Ju Yeo, Ik-Soon Jang

**Affiliations:** 1 Drug & Disease Target Group, Division of Bioconvergence Analysis, Korea Basic Science Institute, Daejeon 305-333, Republic of Korea; 2 Department of Biological Sciences, Sungkyunkwan University, Suwon 16419, Republic of Korea; 3 Korea Bioactive Natural Material Bank, College of Pharmacy, Seoul National University, Seoul 08826, Republic of Korea; 4 Department of Health Sciences and Technology, GAIHST, Gachon University, Incheon 21999, Republic of Korea; 5 Neuroscience Research Institute, Korea University College of Medicine, Seoul 136-705, Republic of Korea; 6 East-West Cancer Center, Daejeon University, Daejeon 302-120, Republic of Korea; 7 Department of Biochemistry, College of Medicine, Gachon University, Incheon 21999, Republic of Korea; 8 Division of Analytical Science, University of Science and Technology, Daejeon 34113, Republic of Korea

**Keywords:** Nectandrin B, cellular senescence, reactive oxygen species, AMP-activated protein kinase, human diploid fibroblasts

## Abstract

Nectandrin B (NecB) is a bioactive lignan compound isolated from *Myristica fragrans* (nutmeg), which functions as an activator of AMP-activated protein kinase (AMPK). Because we recently found that treatment with NecB increased the cell viability of old human diploid fibroblasts (HDFs), the underlying molecular mechanism was investigated. NecB treatment in old HDFs reduced the activity staining of senescence-associated β-galactosidase and the levels of senescence markers, such as the Ser^15^ phosphorylated p53, caveolin-1, p21^waf1^, p16^ink4a^, p27^kip1^, and cyclin D1. NecB treatment increased that in S phase, indicating a enhancement of cell cycle entry. Interestingly, NecB treatment ameliorated age-dependent activation of AMPK in old HDFs. Moreover, NecB reversed the age-dependent expression and/or activity changes of certain sirtuins (SIRT1−5), and cell survival/death-related proteins. The transcriptional activity of Yin-Yang 1 and the expression of downstream proteins were elevated in NecB-treated old HDFs. In addition, NecB treatment exerted a radical scavenging effect *in vitro*, reduced cellular ROS levels, and increased antioxidant enzymes in old HDFs. Moreover, NecB-mediated activation of the AMPK pathway reduced intracellular ROS levels. These results suggest that NecB-induced protection against cellular senescence is mediated by ROS-scavenging through activation of AMPK. NecB might be useful in ameliorating age-related diseases and extending human lifespan.

## Introduction

Although more people are living longer, the number of patients with complex aging-related diseases, like cancer, metabolic diseases, and neurodegenerative disorders, are increasing [1]. Because aging itself has long been implicated as a major risk factor for aging-related diseases, the aging process has become an important therapeutic target [2]. Human diploid fibroblast (HDF) is a suitable model for studying the aging process and developing anti-aging strategies. Normal HDFs replicate a finite number of times *in vitro* and then enter a state of terminal growth arrest, termed as cellular senescence [3]. Senescent cells can be identified by the enhanced expression of senescence markers, such as senescence-associated β-galactosidase (SA-β-gal), senescence-associated secretory phenotype, senescence-associated heterochromatin foci, and increased expression of cell cycle-related proteins, including p21^waf1^, p53, p16^Ink4a^, and cyclin D1/2 [4]. Cellular senescence is closely linked with aging as well as the development and progression of aging-associated diseases. Reduced expression of senescence markers can reverse cellular senescence, resulting in extended lifespan and delayed development of aging-associated diseases [5]. In addition, the aging process and age-related diseases can be modulated by regulating the AMP-activated protein kinase (AMPK), sirtuin, and mechanistic target of rapamycin (mTOR) complex 1 (mTORC1) pathways [6–9].

AMPK, a heterotrimeric serine/threonine protein kinase, is composed of catalytic α subunit and regulatory β and γ subunits. The binding of AMP to the γ subunit activates AMPK by promoting Thr^172^ phosphorylation of the catalytic α subunit by liver kinase B1 (LKB1) [10]. Thr^172^ phosphorylation of AMPK can be caused by other serine/threonine kinases, such as Ca^2+^/calmodulin-dependent protein kinase β and transforming growth factor-β-activated kinase 1, and inhibited by protein phosphatases. It can also be inactivated when the catalytic α subunit is phosphorylated on Ser^485^ by other upstream kinases, such as Akt and protein kinase A [11]. AMPK links energetics to longevity [12]. AMPK activation was shown to extend lifespan by reducing oxidative stress via upregulation of thioredoxin, by repressing endoplasmic reticulum stress and inflammatory disorders, and by inducing autophagic clearance during the aging process [13].

Sirtuins belong to the class III histone deacetylase family and are characterized by a NAD^+^-dependent deacetylase activity [14]. The mammalian sirtuin family encompasses seven isoforms (SIRT1−7), which have been implicated in a wide range of cellular functions, including migration, inflammation, apoptosis, metabolism, stress resistance, and aging [9,15]. Recent data have demonstrated that the activation or enforced expression of sirtuins increases the lifespan of animal models, making sirtuins potential targets for healthy aging [16]. Sirtuins also mediate the beneficial anti-aging effects of caloric restriction [17] and natural products, such as resveratrol [18], resulting in extended human lifespan. mTOR is an evolutionarily conserved serine/threonine protein kinase that influences organismal lifespan in various species, ranging from yeast to mammals [9,19]. mTOR exists in two complexes, mTORC1 and mTORC2, which consist of distinct sets of protein binding partners [20]. mTORC1 is sensitive to rapamycin and regulates protein synthesis and cell growth, which are mediated mostly through phosphorylation of p70 ribosomal S6 kinase 1 (p70S6K1) on Thr^389^ and initiation factor 4E-binding protein 1 (4E-BP1) on Thr^37/46^ [21,22]. The PI3K/Akt pathway is a classic upstream pathway of mTORC1 signaling, and the tuberous sclerosis protein 1 and 2 (TSC1/2) complex is an upstream negative regulator of mTOR. Akt phosphorylates and inactivates TSC2 [23], but AMPK phosphorylates and activates TSC2 [24]. AMPK also appears to provide a switch linking mTORC1-p70S6K1 regulation to cellular energy metabolism via phosphorylation of mTOR at Thr^2446^ [25] and the mTOR binding partner Raptor at Ser^722^ and Ser^792^ [26].

Phytochemicals are being increasingly recognized in the field of healthy aging as potential therapeutics against diverse aging-related diseases. *Myristica fragrans* (nutmeg), an aromatic evergreen tree cultivated in India, South Africa, and other tropical countries, has been used in food and is a source of spices. Nutmeg extract and its active constituents, tetrahydrofuroguaiacin B, nectandrin A (Nec A), and nectandrin B (NecB), have been suggested for use in the treatment of obesity, type-2 diabetes, and other metabolic disorders, presumably via AMPK activation in animal model [27]. Therefore, in this study, NecB was selected as a candidate for preventing aging and age-related diseases. Its effect on cellular senescence in HDFs was examined and the underlying molecular mechanism was clarified by focusing on the AMPK, sirtuin, and mTOR signaling pathways.

## RESULTS

### NecB increases the cell viability of young and old HDFs

**Figure 1:**
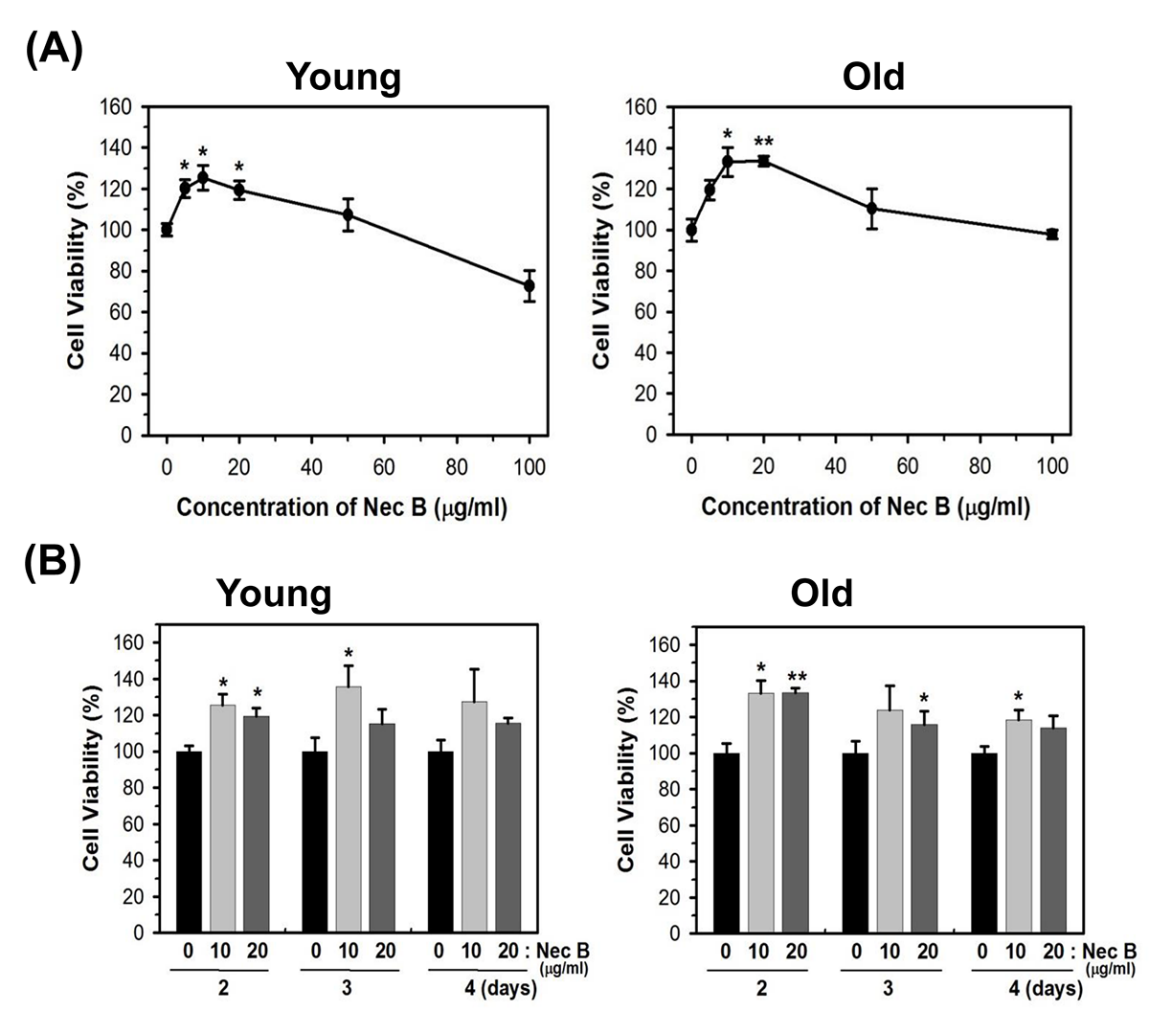
**NecB treatment increased the cell viability of young and old HDFs.** (**A**) Young (PD of 20) and near-senescent (PD of 72, old) HDFs were treated with vehicle (PEG) or various concentrations of NecB for 2 days and the cell viability was assessed by MTT assay. (**B**) Young and old HDFs were treated with 10 and 20 μg/mL NecB for 2−4 days and the cell viability was assessed by MTT assay. *P<0.05, **P<0.01, compared with vehicle-treated control.

HDFs were allowed to undergo numbers of population doubling (PD) to induce replicative senescence. Induction of replicative senescence in cells was validated by positive SA-β gal staining. Because it was not easy to acquire sufficient number of senescent cells, we used near-senescent cells, also termed as old cells. Old cells had cell populations with slower replication capacity and about 30% 5-bromo-4-chloro-3-indolyl-β-d-galactopyranoside (X-gal) staining. Because we observed a similar effect on cell viability by Nec A and NecB, and *Myristica fragrans* (nutmeg) possesses more NecB than Nec A, we utilized NecB for this study. The structure of NecB was identified as 4,4'-[(2R,3R,4S,5S)-3,4-dimethyltetrahydrofuran-2,5-diyl]bis(2-methoxyphenol). To examine the effect of NecB on cellular senescence, young (PD of 20) and near-senescent (PD of 72, old) HDFs were treated with vehicle (polyethylene glycol-300, PEG) or various concentrations of NecB for 2 days and the cell viability was assessed by 3-(4,5-dimethylthiazol-eyl)-2,5-diphenyltetrazolium bromide (MTT) assay. As shown in Fig. 1A, treatment with NecB for 2 days increased cell viability at low concentrations (5−50 μg/mL) but decreased it at higher concentrations (100 μg/mL) in young and old HDFs. The pattern was similar in cells treated with 10 and 20 μg/mL NecB for 3−4 days (Fig. 1B).

### NecB treatment reduces cellular senescence via reduction of senescence marker expression

**Figure 2:**
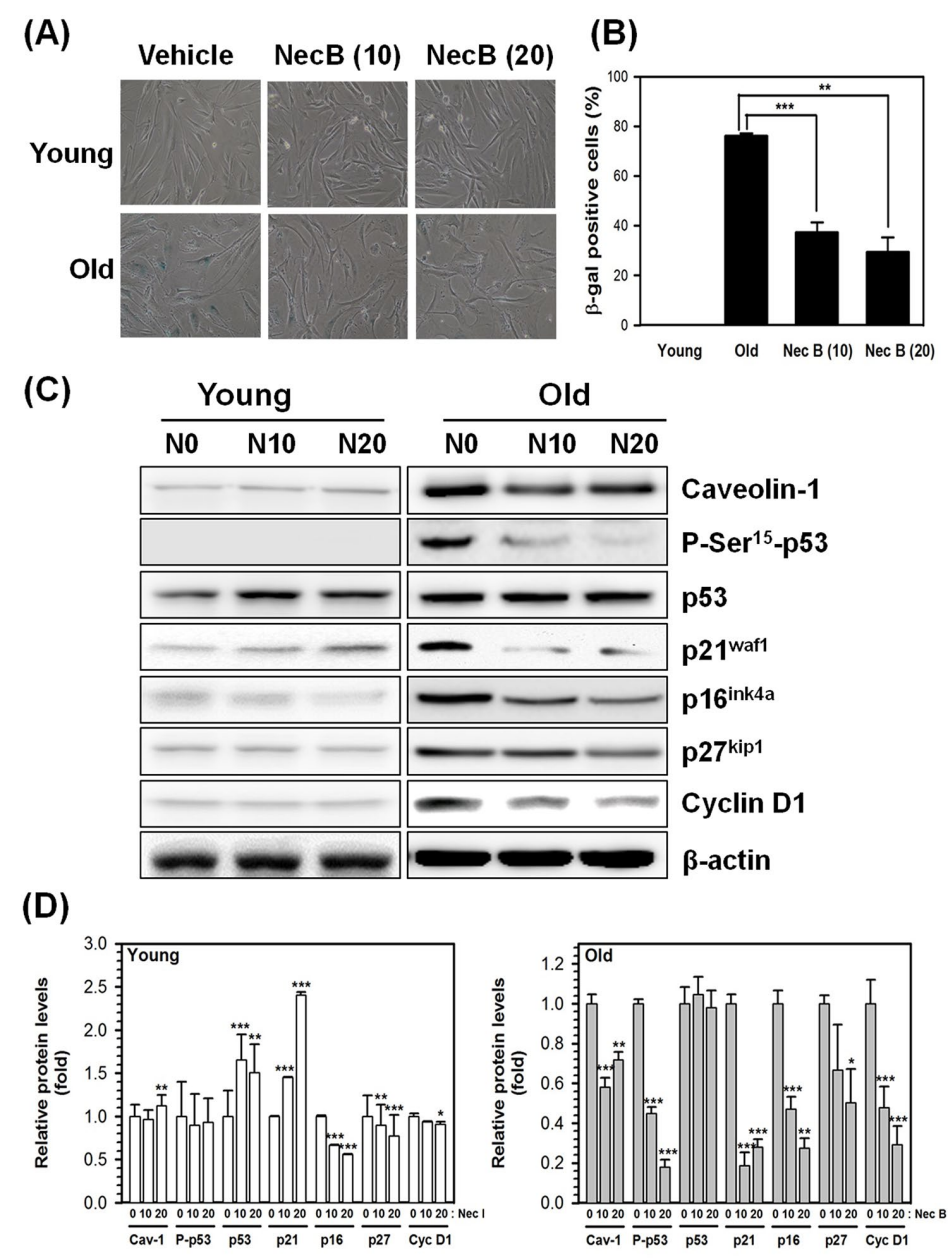
**NecB treatment reduced SA-β-gal activity and senescence marker expression in old HDFs.** (**A**) Young (Y) and old (O) HDFs were treated with vehicle or 10−20 μg/mL NecB for 2 days and stained with X-gal to examine the activity of SA-β-gal. The stained cells were photographed under an inverted microscope (100× magnification). (**B**) The number of SA-β-gal-stained cells in A was counted and its percentage was plotted as the mean ± SEM. **P < 0.01, ***P < 0.001, compared with vehicle-treated control. (**C**) The protein levels in vehicle-treated (N0), 10 and 20 μg/mL NecB-treated (N10 and N20, respectively) young and old HDFs were compared by western blot analysis for senescence markers, including caveolin-1, P-Ser^15^-p53, p53, p21^waf1^, p16^ink4a^, p27^kip1^, and cyclin D1. β-actin was used as an internal control. (**D**) The band density was examined by densitometry and normalized to β-actin. The relative protein levels were calculated and plotted as the mean ± SEM.

SA-β-gal is a lysosomal enzyme with an optimum pH of 4.0−4.5. It is one of the most commonly used markers for cellular senescence. In senescent HDFs, the SA-β-gal activity has been shown to increase and can be detected at pH 6 [3]. To confirm whether the increase in cell viability by NecB in old cells might be caused by reduction of cellular senescence, the activity of SA-β-gal was assessed by X-gal staining of young and old HDFs treated with either vehicle or NecB (10 or 20 μg/mL) for 2 days. As shown in photographs (Fig. 2A) and a plot of the percentage of SA-β-gal positive cells (Fig. 2B), NecB significantly (P<0.001 at 10 μg/mL NecB and P<0.01 at 20 μg/mL NecB) reduced the number of SA-β-gal-stained cells in old HDFs.

In addition to SA-β-gal, caveolin-1 and cell cycle-related proteins, such as p53, p21^waf1^, and p16^ink4a^, are markers of cellular senescence. To confirm the effect of NecB on cellular senescence, we examined the expression of these senescence markers by western blot analysis. β-actin was used as an internal control. As shown in Fig. 2C and 2D, NecB increased the protein level of total p53 and p21^waf1^, but not of P-Ser^15^-p53 in young HDFs. The expression of caveolin-1, P-Ser^15^-p53, p21^waf1^, p16^ink4a^, p27^kip1^, and cyclin D1 was increased in old HDFs. The increase in protein levels was reduced by NecB treatment (10−20 μg/mL) in old HDFs. Alteration in the phosphorylation status of p53 was correlated with changes in p21^waf1^ expression. These data suggest that NecB prevents cellular senescence via reduced expression of senescence markers, such as caveolin-1 and P-Ser^15^-p53, and cell cycle-dependent protein kinase inhibitors, including p21^waf1^, p16^ink4a^, and p27^kip1^ (Fig. 2C and 2D).

### NecB treatment reduces cell cycle arrest and cell death in old HDFs

**Figure 3:**
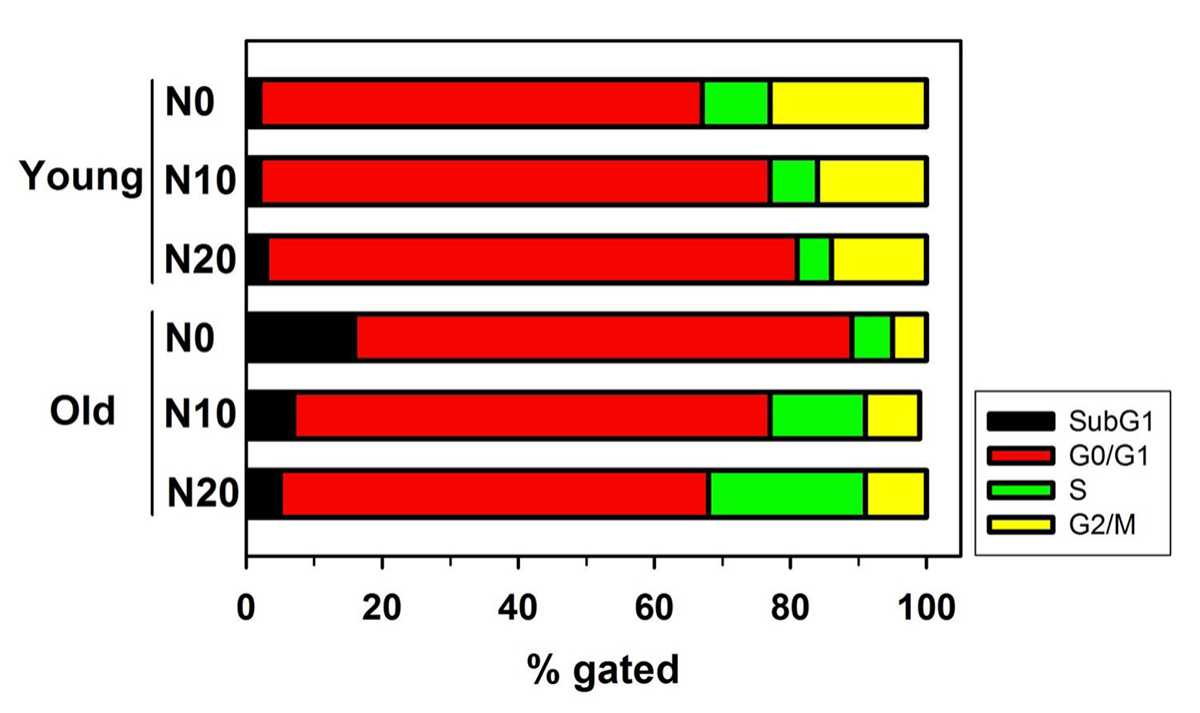
**Flow cytometry for analysis of cell cycle and apoptosis.** Graph shows the relative percentage of cells of the indicated genotype in the subG1, G0/G1, S, and G2/M phases of the cell cycle. Young and old HDFs were treated with either vehicle or NecB (10 or 20 μg/mL) for 16 h, stained with PI, and then analyzed by flow cytometry.

After NecB treatment, alterations in cell cycle and cell death were examined in HDFs. Young and old HDFs were treated with either vehicle or NecB (10 or 20 μg/mL) for 16 h. The cells were then stained with propidium iodide (PI) and analyzed by flow cytometry. The percentage gating for each fraction was compared among groups. Aging itself enhanced the number of cells in sub-G1 fraction (from 2% to 16%) as well as G0/G1 fraction (from 65% to 73%), indicating an increase in cell death and cell cycle arrest (Fig. 3, Young vs. Old). In young HDFs, NecB treatment increased the number of cells in G0/G1 arrest from 65% to 75% and 78%, but reduced that in G2/M fraction from 23% to 16% and 14%, at 10 and 20 μg/mL NecB, respectively (Fig. 3, Young). However, NecB treatment of old HDFs for 16 h reduced the number of cells in sub-G1 phase (from 16% to 7% and 5% at 10 and 20 μg/mL NecB, respectively) and G0/G1 phase (from 73% to 70% and 63% at 10 and 20 μg/mL NecB, respectively) and increased the number of S phase cells (from 6% to 14% and 23% at 10 and 20 μg/mL NecB, respectively). These data suggested that NecB protects old HDFs from cell death and cell cycle arrest, thereby enhancing the rate of cell cycle progression into S phase for DNA synthesis.

### NecB treatment reduces age-dependent AMPK activation in old HDFs

Because the expression of P-Ser^15^-p53, a substrate of AMPK, was increased in old HDFs, and NecB was shown to stimulate the AMPK-related pathways in differentiated C2C12 cells [27–29], the expression and phosphorylation status of AMPK were examined in HDFs. After treatment with vehicle, an AMPK activator AICAR (positive control), an AMPK inhibitor compound C (negative control), or 10−20 μg/mL NecB for 2 days, the protein levels in cell lysates were examined by western blot analysis for AMPK phosphorylation on Thr^172^ (P-Thr^172^-AMPK; an activated form of AMPK) and total AMPK, using glyceraldehyde-3-phosphate dehydrogenase (GAPDH) as an internal control.

**Figure 4:**
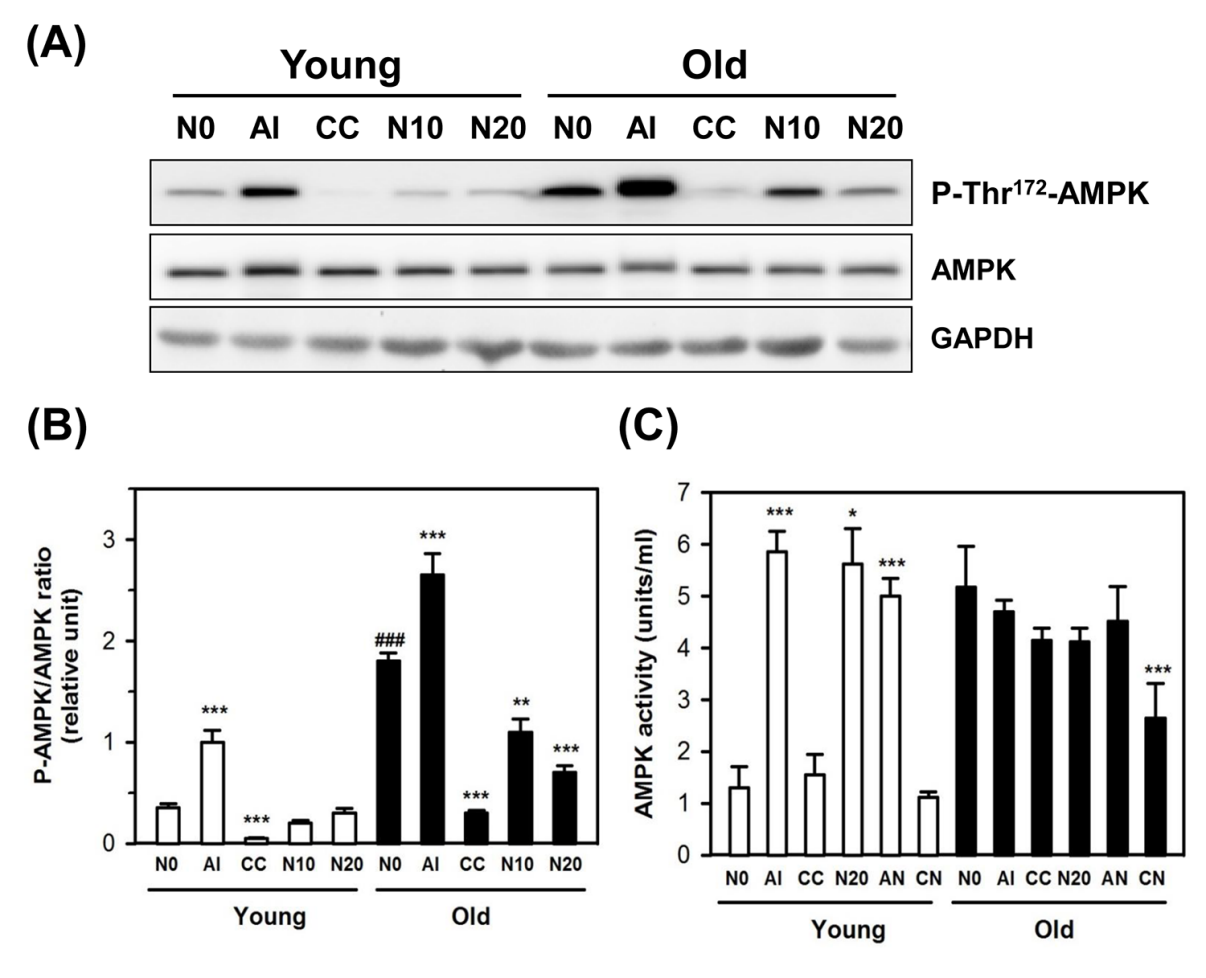
**Effect of NecB on the expression and activation of AMPK in young and old HDFs.** (**A**) Young and old HDFs were treated with either vehicle (N0), or AICAR (AI), compound C (CC), or 10−20 μg/mL NecB (N10 and N20) for 2 days and the cell lysates were analyzed by western blot for AMPK phosphorylation on Thr^172^ and total AMPK, using GAPDH as an internal control. (**B**) The band density was analyzed by densitometry and plotted as the ratio of P-AMPK/total AMPK. (**C**) AMPK activities in vehicle-, AI-, CC-, or NecB-treated cells were assessed as described in Materials and Methods, and plotted as units/mL. *P < 0.05, **P < 0.01, and ***P < 0.001, compared with vehicle-treated control.

In the present study, we observed that although the total AMPK level was not altered by aging, the level of P-Thr^172^-AMPK was increased, which was reduced by treatment with NecB in old HDFs (Fig. 4A and B). The level of P-Thr^172^-AMPK was increased significantly by the AMPK activator NecB and was reduced by the AMPK inhibitor compound C in young and old HDFs. The AMPK activity assay also showed an increase in AMPK activity as a result of aging itself, and AMPK activation by NecB was abrogated in old HDFs (Fig. 4C). However, age-dependent AMPK activation was reduced slightly by compound C or NecB alone, even though there was no significant difference. When old HDFs were co-treated with NecB and compound C, the AMPK activity was significantly (P<0.001) reduced (Fig. 4C). Our data suggest that NecB treatment resulted in reduced Thr^172^ phosphorylation of AMPK, thereby reducing its activity in old HDFs.

### NecB treatment alters the expression and/or activity of some sirtuins, protein synthesis regulators, and cell survival/death-related proteins in old HDFs

**Figure 5:**
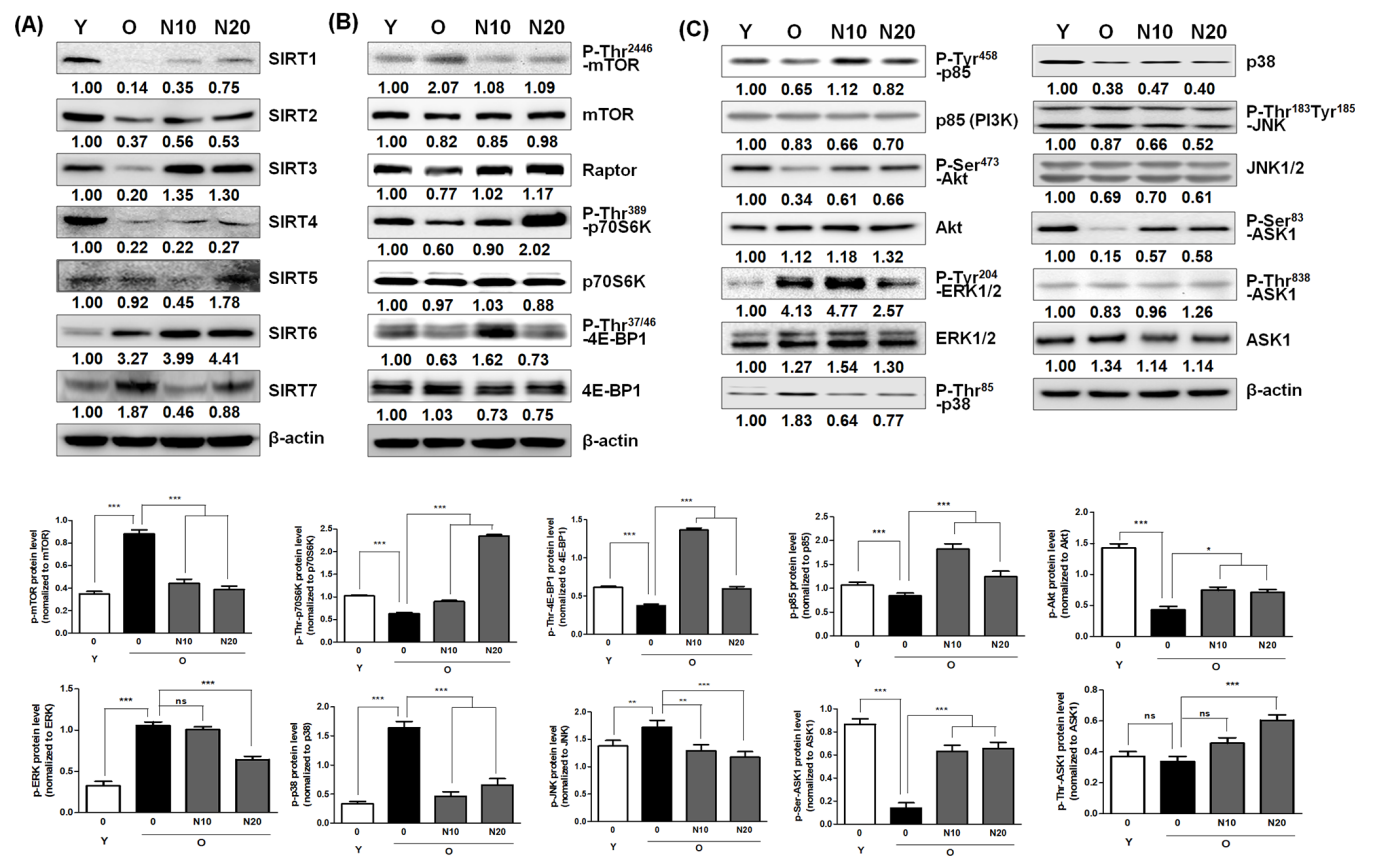
**Effect of NecB on the expression and/or activation of sirtuins, protein synthesis regulators, and cell survival/death-related proteins.** Young and old HDFs (Y and O) were treated with vehicle or 10 or 20 μg/mL NecB (N10 or N20 in old HDFs) for 2 days, The cell lysates were analyzed by western blot with antibodies for sirtuins (**A**), such as SIRT1−7, protein synthesis regulators (**B**), including P-Thr^2446^-mTOR, mTOR, raptor, P-Thr^389^-p70S6K, p70S6K, P-Thr^37/46^-4E-BP1, and 4E-BP1, and cell survival/death-related proteins (**C**), including P-Tyr^458^-p85, p85 (PI3K), P-Ser^473^-Akt, Akt, P-Tyr^204^-ERK1/2, ERK1/2, P-Thr^85^-p38, p38, P-Thr^183^Tyr^185^-JNK, JNK1/2, P-Ser^83^-ASK1 (inactive form), P-Thr^845^-ASK1 (active form), and total ASK1. β-actin was used as an internal control.

Previously, it was shown that SIRT1 expression decreased significantly with multiple passage both in human and murine cells, and a significant positive correlation between SIRT1 level and cell proliferation, and an inverse association between SIRT1 and SA-β-gal activity was reported [30]. Therefore, the effect of NecB on the expression of various sirtuins (SIRT1−7) was examined by western blot analysis in young and old HDFs. The protein levels of SIRT1−7 in vehicle- or NecB-treated old HDFs were then compared with those in young HDFs. As shown in Fig. 5A, aging caused a reduction in the levels of SIRT1−5, but increased the levels of SIRT6 and SIRT7 in the HDF culture system. Treatment with 10−20 μg/mL NecB increased the expression of SIRT1−5, but reduced the enhanced expression of SIRT7. The level of SIRT6 was not altered by NecB treatment.

The mTOR complex is considered one of key regulators of aging and aging-related diseases [9,19]. p70S6K and initiation factor 4E-BP1 are two major protein synthesis regulators, and are the main targets of mTORC1 [21,22]. Therefore, the effect of NecB on the expression levels of protein synthesis regulators, including the two components of mTORC1 (mTOR and raptor), p70S6K, and 4E-BP1 was examined by western blot analysis. The level of P-Thr^2446^-mTOR (inactive and AMPK-dependent) was enhanced by aging, and then reduced after NecB treatment for 2 days (Fig. 5B). Similarly, P-Thr^389^-p70S6K and P-Thr^37/46^-4E-BP1 (inactive, p70S6K-dependent) levels were reduced by aging; however, this effect was reversed by NecB treatment. These NecB-dependent alterations can cause increased protein synthesis. An inverse correlation between P-Thr^2446^-mTOR and P-Thr^389^-p70S6K or P-Thr^37/46^-4E-BP1 levels was detected after NecB treatment. In old HDFs, the expression of an mTOR-associated factor, raptor, was slightly reduced, which was recovered by NecB treatment. Our data suggest that NecB treatment could alter protein synthesis due to its effect on protein synthesis regulators in old HDFs.

AMPK is inactivated by the phosphorylation of catalytic α subunit on Ser^485^ via Akt activation [11]. Therefore, we further examined the effect of NecB on cell survival/death-related proteins, including PI3K and Akt. Moreover, MAPKs are associated with both cellular proliferation and oxidative stress-induced aging. Therefore, we also examined the effect of NecB on MAPKs, including extracellular signal-regulated kinase 1/2 (ERK1/2), p38, c-Jun N-terminal kinase 1/2 (JNK1/2), and apoptosis signal-regulating kinase 1 (ASK1). Young and old HDFs were treated with vehicle or 10 or 20 μg/mL of NecB for 2 days. The cell lysates were analyzed by western blotting. The phosphorylated protein levels of p85PI3K (P-Tyr^475^-p85) and Akt (P-Ser^473^-Akt) were reduced in old HDFs, but NecB treatment increased them (Fig. 5C), resulting in the activation of PI3K and Akt. In contrast, the phosphorylation and activity of ERK1/2 and p38 were enhanced by aging and NecB treatment reversed these aging-dependent alterations in old HDFs (Fig. 5C). There was no change in the levels of total and phosphorylated JNK1/2. Further, the level of the phosphorylated form of ASK1 (P-Ser^83^-ASK1; inactive form) was enhanced by NecB treatment, indicating inactivation of the stress-associated ASK1-p38 pathway. These data suggest that NecB treatment enhanced aging-induced reduction of cell growth and survival, but reduced aging-induced cellular stress and death.

### NecB treatment increases the transcriptional activity of YY1 and the expression of downstream proteins including Snail in old HDFs, but not in young HDFs.

**Figure 6:**
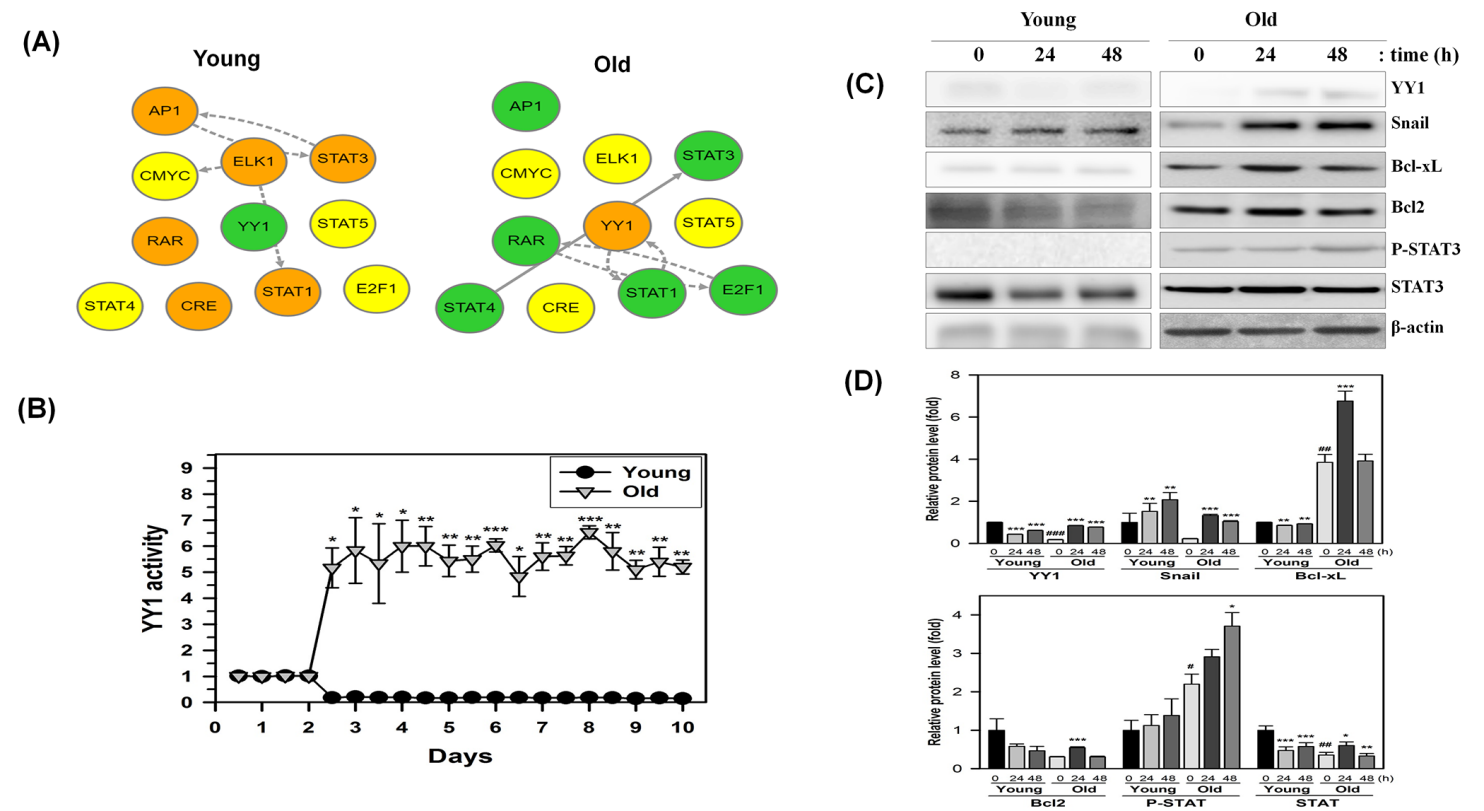
**Time-dependent changes in the transcriptional regulatory network in NecB-treated young and old HDF cells.** (**A** and **B**) After NecB treatment of young and old HDFs seeded in a 96-well plate, the expressions of several transcription factors were investigated by measuring the fluorescence intensity every 12 h for 10 days in cells transduced with a lentiviral vector containing transcription factor binding sites. The transcriptional regulatory network was inferred by BTNET. The color of each circle in A indicates the expressional change of each transcription factor (red for up-regulation and green for down-regulation) and the arrow between circles indicates edge information (dotted line for inference and solid line for prior knowledge). The time-dependent activation of transcription factor YY1 in NecB-treated young and old HDFs was plotted in B. (**C**) Young and old HDFs were treated with vehicle or 10 μg/ml NecB for 24-48 h. Cell lysates were analyzed by western blotting with antibodies against YY1, Snail, Bcl-xL, Bcl2, P-STAT3, STAT3, and β-actin. (**D**) The band density of the immunoblot was quantitated by using Image J software and normalized to β-actin. The relative protein levels (fold) were calculated and plotted as the mean ± SEM.

Recent studies have revealed that AMPK downregulates mTORC1 activity [24,26] and the mTORC1 activity was implicated in modulation of a transcription factor Yin-Yang 1 (YY1), which plays a role in proliferation and cell death [8]. Therefore, we postulated that YY1 and downstream proteins might be involved in NecB-induced anti-senescence effects in old HDFs. We first examined the transcriptional networks including YY1 in young and old HDFs after NecB treatment. As shown in Fig. 6A, NecB reduced YY1 transcriptional activity in young cells, whereas it enhanced that in old cells. In addition to YY1, NecB differentially regulates various transcription factors, such as AP1, E2F1, CRE, ELK1, STATs (1, 3 and 4), and RAR between young and old HDFs. NecB treatment increased the transcriptional activity of YY1 from 2.5 days until 10 days in old HDFs but not in young HDFs (Fig. 6B). The transcriptional activation of YY1 would increase the levels of downstream proteins, such as Snail, Bcl-xL, Bcl2, and STAT3. Induction of these downstream proteins was confirmed by western blot analysis. The expression of Snail as well as YY1 was induced by NecB treatment at 24-48 h and the level of Bcl-xL Bcl2, and STAT3 was induced transiently at 24 h and returned to the basal level in old HDFs (Fig. 6C and 6D). The phosphorylated STAT3 increased gradually until 48 h. Activation of YY1 and increased expression/activation of downstream proteins by NecB treatment might induce the recovery of impaired mitochondrial function in old HDFs, resulting in cell proliferation and survival.

### NecB treatment reduces intracellular ROS production in old HDFs through activation of AMPK

**Figure 7:**
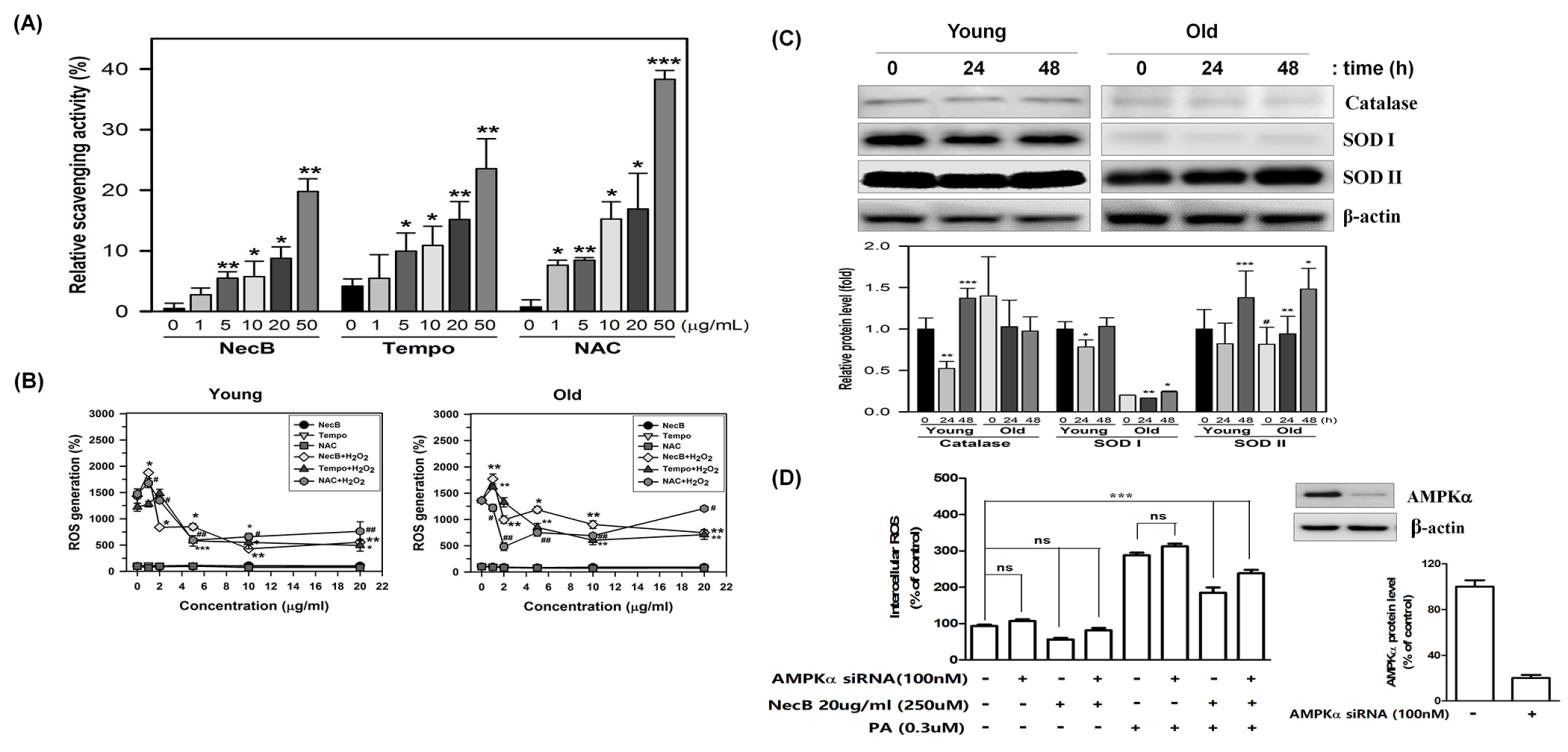
**Radical scavenging activity and anti-oxidative effect of NecB.** (**A**) Radical scavenging effects of various concentrations of NecB and two antioxidant controls, Tempo and NAC, were examined by incubation with DPPH reaction mixture, in which DPPH radical was induced by sonication. Percent scavenging activities of NecB were calculated and are plotted as the means ± standard deviations of at least three experiments. *P<0.05, **P<0.01, and ***P<0.001 compared with vehicle-treated control. (**B**) Young and old HDFs were pretreated with vehicle (PBS) or 100 μM H_2_O_2_ for 4 h and then treated with NecB, Tempo, or NAC for 24 h. The ROS level was measured by multiple plate reader after staining with DCF-DA. (**C**) Young and old HDFs were treated with NecB for 0, 24, or 48 h and the cellular levels of antioxidant enzymes (catalase, SOD I and SOD II) and β-actin were assessed by western blotting. The band density of the immunoblot was examined by densitometry and normalized to β-actin in each lane. The relative protein levels were calculated and plotted as the mean ± SEM. (**D**) Activation of the AMPK pathway reduced intracellular ROS levels A: AMPK was involved in the Nectandrin B-induced reduction in ROS levels. HDFs were transfected with AMPK siRNA and then treated with Nectandrin B in the absence or presence of palmitic acid for 24 h. The effectiveness of AMPKα knockdown was examined by anti-AMPKα antibody. ROS were detected by DPPH assay. Data represent the means ± SE (n = 3). **P < 0.01 ***P < 0.001. AMPKα siRNA prevented Nectandrin B-induced reduction of ROS.

Aging itself and aging-associated signaling pathways, such as the AMPK, sirtuins, and mTOR pathways, are closely related with oxidative stresses. Therefore, it was determined whether the effect of NecB on cellular senescence can be caused by radical scavenging and antioxidant effects. The radical scavenging effect was examined using the *in vitro* assay system with 2,2-diphenyl-1-picrylhydrazyl (DPPH) compound composed of stable free-radical molecules. The direct radical scavenging activity of well-known antioxidants, such as Tempo and N-acetyl-cysteine (NAC) was also measured and compared to that of NecB. The *in vitro* DPPH assay indicated that NecB exerted a radical scavenging effect *in vitro* to a similar extent to those of Tempo and NAC (Fig. 7A).

Additionally, we evaluated whether NecB treatment decreases intracellular ROS levels. To measure the effect of NecB on intracellular ROS, HDFs were pretreated with 100 μM H_2_O_2_ for 4 h and then treated with NecB, Tempo, and NAC (10 μg/mL) for 24 h. The cellular ROS level was quantified after staining with DCFH-DA. Interestingly, NecB treatment significantly reduced H_2_O_2_-induced intracellular ROS production in both young and old HDFs (Fig. 7B). Furthermore, the levels of antioxidant enzymes, such as catalase and superoxide dismutase (SOD) I and II were investigated by western blot analysis in young and old HDFs. The results showed that NecB treatment differentially stimulated the expression of these antioxidant enzymes between young and old HDFs (Fig. 7C). NecB treatment slightly reduced the level of catalase at 24 h but enhanced it at 48 h in young cells. However, the level of catalase was not significantly altered in old cells. In contrast, NecB treatment did not altered the levels of SOD I and II, whereas it significantly up-regulated them in old HDFs (Fig. 7C).

AMPK reduced ROS levels induced by palmitic acid in HDFs. We ﬁrst tested whether activation of the AMPK pathway could reduce palmitic acid-induced ROS production. HDFs were incubated with increasing amounts of NecB in the presence or absence of palmitic acid; ROS levels were detected in the treated cells. As shown in Fig. 7D, NecB treatment alone had minimal effects on basal ROS levels. Palmitic acid signiﬁcantly increased intracellular ROS levels. The palmitic acid–induced increase in intracellular ROS levels was reduced by NecB in a dose-dependent manner with up to a 60% reduction at the highest dose (20ug/ml). This result indicates that activation of AMPK can reduce intracellular ROS levels. Additionally, suppression of AMPK by speciﬁc siRNAs not only increased basal ROS levels, but also augmented the palmitic acid–induced increase in ROS levels (Fig. 7D). Furthermore, the NecB-induced reduction in ROS levels was abolished by AMPK siRNA. Taken together, these data suggest that NecB protects cellular senescence by reducing cellular oxidative stress via the direct radical scavenging effect or indirectly via the induction of antioxidant enzymes, such as SOD I and II through activation of AMPK.

## DISCUSSION

Previous studies have shown that replicative senescence of HDFs leads to whole body aging and age-related diseases, such as cancer, stroke, infarction, Alzheimer’s disease and Parkinson’s disease, and diabetes type 2, which eventually limit lifespan [31]. Therefore, it has been postulated that drugs preventing cellular aging might delay whole body aging and potentially be used in the treatment of age-related diseases [2]. Interestingly, NecB treatment reversed aging-induced changes, such as overexpression of typical senescence markers, such as SA-β-gal activity, P-Ser^15^-p53, p21^waf1^, p16^ink4a^, p27^kip1^, caveolin-1, and cyclin D1 (Fig. 2), cell cycle arrest at the G0/G1 phase and apoptotic cell death (Fig. 3), and overexpression of stress-induced apoptosis markers, such as ERK1/2 and p38, and reduction of survival factors, including PI3K, P-Akt, and P-Ser^83^-ASK1 (Fig. 5). Inactivation of these stress-associated pro-apoptotic pathways and upregulation of survival factors might be responsible for the reduction of apoptosis by NecB in old HDFs. NecB treatment could reduce the number of senescent cells, thereby decreasing the pathology of organ aging. Our data suggest the possibility that NecB can be selected as a therapeutic candidate for preventing aging-related diseases. Since the SIRT1, AMPK, and mTOR pathways have been implicated in aging and age-related diseases [13,18,32], we focused on these three pathways to elucidate the molecular mechanism of NecB effect on old HDFs.

Previously, NecB was suggested as an AMPK activator in various cell types, including human HDFs [27], hepatocytes [33], rat VSMCs [29], and differentiated C2C12 cells [28]. It was also shown that the activation capacity of AMPK declines during aging [13]. Overexpressing and activating AMPK were sufficient to extend lifespan in various model organisms [34,35]. Therefore, the search for AMPK activators for use as longevity inducers produced a range of small molecules, such as metformin, resveratrol, rapamycin, and aspirin [12]. However, many of these effects are indirect, and their working mechanisms to promote healthy aging have not been elucidated. Previously, NecB was shown to exert a hepato-protective effect against oxidative damage indirectly via Nrf2 activation and subsequent expression of an array of antioxidant enzymes [33]. The activation of AMPK might suppress oxidative stress-induced cellular senescence [36].

Since oxidative stress increases with aging and AMPK is activated by oxidative stress [37], the basal level of P-Thr^172^-AMPK can be elevated as cells undergo senescence [38]. As expected, our data showed an enhancement of AMPK phosphorylation and activity in old HDFs (Fig. 4). Although NecB was suggested as an AMPK activator, treatment of old cells with NecB reduced the activity of AMPK (Fig. 4). Therefore, it is questioned how NecB exerts its proliferative and anti-aging effect on old HDFs. Since AMPK activation was shown to slow down cell cycle progression presumably via p53 phosphorylation on Ser^15^ and transcriptional activation, subsequently resulting in overexpression of p21^waf1^ [39], inactivation of AMPK may be beneficial for cell growth and proliferation in near-senescent cells. In the present study, we also observed the anti-proliferative effect of NecB on young HDFs treated  with a high concentration (100 μg/mL) of NecB (Fig. 1A). Our data suggest that the effect of NecB might be cell type-specific and might differ depending on the drug concentration and cell age.

The mTORC1 activity is known to modulate YY1 transcription factor, which is implicated in cell proliferation and cell death [8]. YY1 can also promote Akt phosphorylation and activation via mTOR activation [40]. In the present study, we showed that NecB treatment significantly increased the protein kinase activities of mTORC1 and Akt (Fig. 5B) and the transcriptional activity of YY1 (Fig. 6) in old but not in young HDF cells. The expression of downstream proteins, such as Snail, Bcl-xL, Bcl2, and STAT3, was also induced in NecB-treated old HDFs. Snail is a zinc finger transcription factor that induces cell movement and survival [41] and regulates cellular senescence in IMR90 normal fibroblast cells [42]. The activated YY1 may be recruited to the antioxidant responsive elements (AREs) binding site and then amplified the NRF2-mediated ARE transcription and subsequent cell protection against oxidative damage [43]. Indeed, we observed the induction of antioxidant enzymes, such as SOD I and II (Fig. 7CD). In the meanwhile, NecB reduced cellular oxidative stress via the direct radical scavenging effect (Fig. 7AB). The data suggest that YY1 may play a role in NecB-induced proliferation of aged cells by reducing cellular oxidative stress through the direct radical scavenging effect or indirectly through the induction of antioxidant enzymes via YY1 activation. In addition, ROS scavenging effect of NecB might cause a reduction of AMPK activity in old HDFs (Fig. 4).

A previous study showed that SIRT1 expression decreased significantly with serial cell passages in both human and murine cells [30]. Enforced SIRT1 expression promoted cell proliferation and antagonized cellular senescence [44]. Depletion of SIRT1 induced aging-like phenotypes [45]. SIRT1 also plays a role in delaying ultraviolet B (UVB)-induced skin aging by suppressing oxidative stress and p53 acetylation in skin fibroblasts [46]. Similarly, the levels of SIRT3, a mitochondrial deacetylase, were decreased in high glucose-induced senescent cells [47] and in the aged brain of mice [48]. Reduced levels of SIRT3 lead to increased ROS production and subsequent aging via increase in activated p53 level [49]. In addition, SIRT3 has a pro-proliferative function in human melanoma cells [50]. Because we observed a reduction in the levels of SIRT1 and 3, we suggest that the reduced protective effect and pro-proliferative function by SIRT1 and SIRT3 might contribute to cellular aging in our HDF cell system. Because SIRT4 and SIRT5 are also mitochondrial sirtuin deacetylases, they might function in a manner similar to SIRT3.

Age-related changes in the expression of other sirtuins, including SIRT2, SIRT6, and SIRT7 are species/tissue/organ-specific. SIRT2 is an NAD^+^-dependent tubulin deacetylase that regulates cell cycle progression and longevity or by inducing the checkpoint kinase BubR1 [51]. It has been suggested that overexpression of SIRT2 is a novel marker of aging [48]. However, SIRT2 level was decreased by aging in our experimental model (Fig. 5). Since SIRT2 has been reported to regulate lifespan and longevity in fruit fly and nematodes, but not in humans [52], we postulated that the age-related expression of SIRT2 might be species-dependent. SIRT6 is another important DNA repair-related protein that regulates gene expression in mammalian cells. SIRT6 acts as a safeguard against oxidative stress by co-activating Nrf2 [53]. Interestingly, a decrease in SIRT6 expression was observed in the brain of aging mice [48]. However, we observed a slight increase in the expression of SIRT6 in old HDFs. Because Jung et al. reported that p53 directly activates SIRT6 expression [54], and we observed an increase in p53 activity during aging, we hypothesized that age-induced SIRT6 expression might be caused by p53 activation. Unlike SIRT6, the expression pattern of SIRT7 is in agreement with a previous report [55]. The expression of SIRT7 was higher in old cells than that in young cells (Fig. 5), indicating a possible role of SIRT7 in aging. Taken together, these findings suggest that aging causes an increase in the levels of some sirtuins, including SIRT6 and SIRT7, to maintain tissue integrity against age-related DNA damage by inhibiting cell proliferation and growth.

Due to the protective effects of sirtuins against cellular and organismal aging, chemical agents that up-regulate or activate sirtuins might be beneficial. Resveratrol has been shown to induce anti-oxidant effects and ameliorate age-associated phenotypes by activating SIRT1 [12,36]. A natural phytochemical dehydroabietic acid was shown to activate SIRT1 and SIRT3, resulting in anti-aging effects [56]. In addition, pyrroloquinoline quinine protects against UVA-induced senescence in skin fibroblasts via activation of SIRT1 and SIRT6 [57]. Juglone, which up-regulates SIRT1 in skin cells, was also reported to play some protective roles against normal and UVB-induced skin aging [58]. Although aging causes differential changes depending on the type of sirtuin, NecB treatment resulted in an increase in the levels of almost all sirtuins, including SIRT1‒6 in old HDFs (Fig. 5). Therefore, it can be suggested that NecB exerts its anti-senescence effect similarly via activation of sirtuins and subsequent modulation of ROS and p53 activation.

Because mTORC1-regulated processes promote cellular aging and deactivation of the mTOR pathway slows down the aging process, the mTORC1 pathway has become a new target for anti-aging research and prevention of age-related diseases [9,19]. In a previous report, pretreatment of VSMCs with PI3K and Akt inhibitors, the mTOR inhibitor rapamycin, and mTOR siRNA blocks the changes in replicative senescence marker expression in VSMCs [59]. In another report on peripheral blood mononuclear cells (PBMCs), the basal levels of mTOR mRNA or protein were increased but their sensitivities to growth signals were reduced during cellular senescence [60]. In contrast to the previous reports, in this study, the level of P-Thr^2446^-mTOR (inactive) was increased and that of P-Thr^389^-p70S6K and P-Thr^37/46^-4E-BP1 was reduced by aging (Fig. 5), indicating reduction of the mTOR signaling pathway in old HDFs. NecB treatment reversed these aging-associated alterations in old HDFs (Fig. 5), causing increased protein synthesis and cell proliferation via activation of the mTOR pathway. The NecB-induced effects were different from those induced by resveratrol and caloric restriction at least in terms of activation of mTORC1. Resveratrol and caloric restriction can extend lifespan via suppression of the mTOR pathway, but it is still a subject of debate whether this mechanism is operational in humans as well.

Because a negative cross-talk between the AMPK and mTOR signaling pathways has been proposed in insulin-mediated phosphorylation of p70S6K1 [61], the reduced mTOR signaling in old HDFs could be AMPK-dependent. Moreover, Akt activates mTOR at least in part through direct phosphorylation, and causes a decline in AMPK activity [23]. AMPK activation, presumably via Akt inhibition, may increase the phosphorylation of mTOR at Thr^2446^ in old HDFs, thereby restricting mTOR activation in old HDFs. There is also substantial cross-talk between AMPK and sirtuins [9]. SIRT1 may deacetylate and activate LKB1, leading to increased Thr^172^ phosphorylation of AMPKα and its subsequent activation [32]. Moreover, the crosstalk between sirtuins and mTORC1 signaling has been implicated in the activation of p70S6K [7]. The molecular mechanism by which NecB treatment reduces AMPK activity, increases the expression of certain sirtuins, and enhanced the mTOR signaling pathway in old HDFs remains to be elucidated. Crosstalk between AMPK, sirtuins, and mTOR is context-dependent and might involve many other cellular signaling molecules or cellular components (Fig. 6), resulting in different outcomes depending on cell type and age.

In a previous report, activation of the AMPK-FOXO3 pathway reduces intracellular reactive oxygen species [62,63]. AMPK reduced ROS levels induced by palmitic acid in HDFs. The palmitic acid–induced increase in intracellular ROS levels was reduced by NecB in a dose-dependent manner. This result indicates that activation of AMPK can reduce intracellular ROS levels. Additionally, suppression of AMPK by speciﬁc siRNAs not only increased basal ROS levels, but also augmented the palmitic acid–induced increase in ROS levels (Fig. 7D). Taken together, these data suggest that NecB protects cellular senescence by reducing cellular oxidative stress via the direct radical scavenging effect or indirectly via the induction of antioxidant enzymes through activation of AMPK.

**Figure 8:**
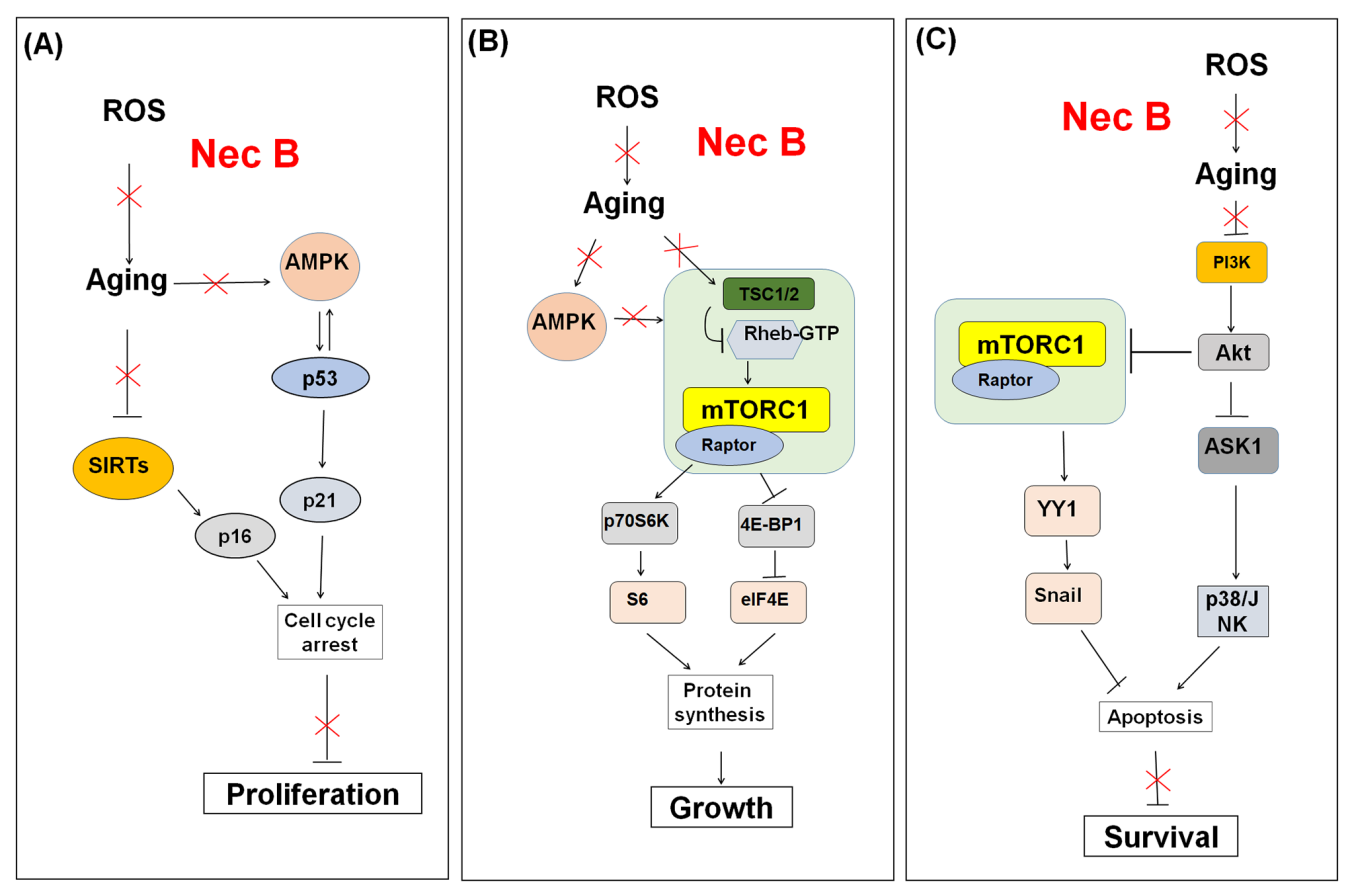
Schematic model for the effect of NecB on (**A**) cell proliferation, (**B**) growth, and (**C**) survival in near-senescent HDFs. The red color X means inhibitory effect by NecB treatment in old HDFs.

In addition, we showed that NecB affected the AMPK, sirtuin, and mTOR signaling pathways in near-senescent old HDFs (Fig. 8). Thus, these pathways are potential targets for NecB-induced protective effects against cellular senescence in HDFs. NecB might be useful in ameliorating age-related diseases, improving health, and extending human lifespan.

## MATERIALS AND METHODS

### Materials

Reagents were purchased from the vendors as described below. Dulbecco’s modified Eagle’s medium (DMEM), MTT, trypan blue, X-gal, PI, PEG, NecB, and compound C were purchased from Sigma-Aldrich (St. Louis, MO, USA). Fetal bovine serum (FBS), penicillin, streptomycin, and other cell culture products were obtained from Life Technologies (Grand Island, NY, USA). Polyclonal antibodies against caveolin-1, phosphorylated P-Ser^15^-p53, p21^waf1^, and p16^ink4a^, total AMPK, P-Thr^172^-AMPK, total mTOR, P-Thr^2446^-mTOR, raptor, total p70S6K, P-Thr^389^-p70S6K, total 4E-BP1, P-Thr^37/46^-4E-BP1, total p85, P-Tyr^458^-p85, total Akt, P-Ser^473^-Akt, total p38, P-Thr^85^-p38, total ASK1, P-Ser^83^-ASK1 (inactive form), and P-Thr^845^-ASK1 (active form) were from Cell Signaling Technology (Danvers, MA, USA). Polyclonal antibodies against total ERK1/2, P-ERK1/2, total c-Jun N-terminal kinase 1/2 (JNK1/2), P-Thr^183^Tyr^185^-JNK, total p53, cyclin D1, GAPDH, and β-actin, and secondary horseradish peroxidase (HRP)-conjugated anti-rabbit and anti-mouse antibodies were purchased from Santa Cruz Biotechnology (Santa Cruz, CA, USA). Nitrocellulose membranes were purchased from the Pall Corporation (Pensacola, FL, USA); bicinchoninic acid (BCA) protein assay kits and the enhanced chemiluminescence (ECL) system from Pierce Biotechnology (Lockford, IL, USA); and protease inhibitor cocktail from Roche (Mannheim, Germany). NecB isolated from the ethanol extract of nutmeg as previously described [28,29] was supplied by the Korea Bioactive Natural Material Bank (KBNMB, Republic of Korea) at Seoul National University and its purity was confirmed to be > 95% by high performance liquid chromatography.

### Cell culture

Primary HDFs were isolated and cultured as described previously [4]. Cells were maintained in DMEM containing 10% FBS and antibiotics. The molecular contents of young cells from the early stage of culture, with PD of less than 25, were compared to those of senescent cells with PD of 70−80. Senescent cells were characterized by morphological changes, reduced proliferation rates, and enhanced SA-β-gal activity. Since it is difficult to obtain sufficient senescent cells for all experiments, we used near-senescent cells, also termed as old cells, which had a PD of 65−75 with slower replication capacity and about 30% X-gal staining. Prior to NecB treatment, cells were grown for 1−2 days to 60−70% sub-confluence in the culture medium.

### Cell viability assay

Cell viability was assessed by the MTT assay [64]. Young and old HDFs were treated with various concentrations of NecB for 2 days. Cells were then incubated with medium containing 5 mg/mL MTT for 3 h at 37 °C. After the medium was discarded, dimethylsulfoxide (DMSO) was added to dissolve the blue formazan crystals. After 10 min of incubation, the absorbance at 570 nm was measured using an enzyme-linked immunosorbent assay (ELISA) reader (Multiskan EX; Thermo Lab systems, Beverly, MA, USA).

### SA-β-gal staining

Young and old HDF cells were fixed and stained for SA-β-gal activity as described previously [4]. Cells were washed twice with phosphate buffered saline (PBS) and then fixed with 2% formaldehyde/0.2% glutaraldehyde in PBS for 10 min. The fixer was removed by washing twice with PBS and cells were incubated with fresh SA-β-gal staining solution containing 1 mg/mL X-gal, 5 mM potassium ferrocyanide, 5 mM potassium ferricyanide, and 2 mM MgCl_2_ in 40 mM citric acid/sodium phosphate buffer, pH 6.0, for 12 h in a CO_2_-free 37 °C incubator. The stained cells were photographed at 100× magnification under an inverted microscope with a camera (Nikon Eclipse TS100, Nikon, Melville, NY, USA).

### Cell cycle analysis

The effect of NecB treatment on cell cycle progression was determined by flow cytometry as described previously [65]. The DNA content was assessed by staining the ethanol-fixed cells with PI. Briefly, HDFs (1×10^6^ cells/mL) were seeded in 60-mm tissue culture dishes, cultured for 1 day, and then treated with vehicle (DMSO) or 10−20 μg/mL NecB. After incubation for the indicated times at 37 °C, the cells were harvested, washed twice with PBS, and fixed in ice-cold 70% ethanol overnight at 4 °C. The fixed cells were pelleted by centrifugation at 1,000 × *g* for 5 min, washed twice with PBS containing 0.1% bovine serum albumin, and then incubated with 1 mg/mL of DNase-free RNase A and 50 μg/mL of PI for 30 min at 37 °C in the dark. The cells were analyzed using a FACS Calibur flow cytometer (Becton Dickinson, San Jose, CA, USA).

### Western blotting

For Western blot analysis, young HDFs (3×10^5^ cells/well) were seeded in 3 mL medium and grown for 1 day in a 6-cm culture dish. Cells were treated with 10−20 μg/mL NecB for the indicated times. HDFs were washed twice with ice-cold PBS and total cell lysates were prepared using ice-cold lysis buffer (25 mM HEPES, pH 8.0, 150 mM NaCl, 1 mM EDTA, 1 mM Na_3_VO_4_, 1 mM NaF, 1% Triton X-100, and protease inhibitor cocktail) for 30 min. Whole cell lysates were centrifuged (12,000 × *g* for 10 min at 4 °C) to remove cell debris. The protein concentrations of the lysates were determined using a BCA protein assay kit, as described by the manufacturer. Cell lysates (45 μg) were analyzed by 8−12% sodium dodecyl sulfate-polyacrylamide gel electrophoresis and western blotting with Protran nitrocellulose filters, a blocking solution containing 5% non-fat dried milk and 0.1% Tween 20, and overnight incubation with various primary antibodies in the blocking solution. Blots were further incubated with HRP-conjugated anti-rabbit or mouse IgGs (1:5000) in blocking solution for 1 h at RT and the immune complexes were visualized using an ECL system. Quantification of each band was performed using Image-J software (National Institutes of Health, Bethesda, MD, USA).

### AMPK activity assay

AMPK activity was determined using an AMPK activity assay kit from Invitrogen (Waltham, MA, USA). Following the manufacturer’s guidelines, the cells were lysed by incubation with cell extraction buffer for 30 min on ice. After centrifugation at 10,000 × *g* for 10 min at 4 °C, the supernatant of lysed cells was collected. Cell lysate and phospho-AMPK  standards were diluted with a standard diluent buffer. Standards and vehicle- or NecB-treated samples were added to the wells in a plate pre-coated with capture antibody, and incubated for 2 h at 25 °C. After reaction with AMPKα detection antibody solution for 1 h, cells in each well were incubated with anti-rabbit IgG HRP solution for 30 min, followed by incubation with stabilized chromogen substrate for 30 min at 25 °C in the dark. The stop solution was then added to each well and the absorbance was measured at 450 nm using an ELISA reader within 2 h after adding the stop solution. AMPK activity was calculated as units/mL by comparing with the standard curve, and plotted as the mean ± standard error mean (SEM).

### DPPH radical scavenging activity assay

NecB was tested for its DPPH radical scavenging activity. The DPPH solution (1.3×10^-7^ mol/L) was prepared in 100% methanol and was sonicated for 5 min to obtain the stable free radical form of DPPH. Two hundred μL of the fresh DPPH reagent was mixed with 10 μL of NecB at various concentrations in a 96-well plate. The reaction mixture was vigorously shaken and incubated for 30 min at room temperature in the dark with shaking. The absorbance of the resulting solution was measured at 517 nm against a methanol blank using a spectrophotometer. Two known antioxidants, tempo and NAC (Sigma-Aldrich, St. Louis, MO, USA), was used to compare the free radical scavenging activity at each concentration. The color change of DPPH reduction was compared by adding DMSO or methanol to DPPH solution. All tests were carried out in triplicate. The radical scavenging activity was measured as a decrease in the absorbance of DPPH and the percentage of radical scavenging activity was calculated according to the following equation:

Radical scavenging activity (%) = [1 - {(A517 nm, sample - A517nm, standard)/A517nm, control}] × 100

### Measurement of intracellular reactive oxygen species (ROS) generation

DCFH-DA was dissolved in DMSO and stored as 100 mM at –20 °C. Cells were pretreated with 1× PBS or 100 μM H_2_O_2_ for 4 h and then treated with NecB, Tempo, or NAC for 24 h. Cells were then incubated with 10 μM DCFH-DA for 30 min at 37 °C. DCF fluorescence was detected by a fluorescence spectrometer Infinite F200 PRO (Tecan Group Ltd. Männedorf, Switzerland) with an excitation wavelength of 485 nm and emission wavelength of 535 nm.

### Cloning of vectors containing transcription factor binding sequences

Each transcription factor binding site-encoding sequence was amplified by PCR. The PCR products were gel purified and inserted into pGreenFire^TM^-Pathway Reporter Lentivectors containing a minimal CMV promoter, green fluorescence protein (GFP) reporter and luciferase reporter (SBI System Biosciences, CA, USA). The double-stranded DNA fragments digested by restriction enzymes (EcoRI and SpeI; NEB, MA, USA) were ligated to the linearized lentivirus vector with T4 ligase. The ligated products were transformed into E. coli DH5α cells (Enzynomics, Daejeon, Korea), and selected by sequencing verification. When a specific transcription factor binds to pGreenFire1^TM^-mCMV- Puro vector including transcription binding sequences, GFP and luciferase can be expressed.

### Virus packaging

Human embryonic kidney (HEK) 293T cells were seeded in a 75 cm^2^ cell culture flask (4 × 10^6^ cells per culture dish) with DMEM containing 10% FBS. The expression vector and lentivirus packaging mixture were transfected into HEK 293T cells with Hillymax (Dojindo, Kumamoto, Japan) and opti-MEM medium (Thermo Fisher Scientific, MA, USA). After transfection for 5 h, the primary mixture was replaced with DMEM medium with 1.0 g/L glucose and 5% FBS, and the supernatant was collected at 48 h after transfection. The collected supernatant containing viral vector was filtered by 0.2 μm-pore size syringe filter and stored at -80°C.

### Live cell analysis of transcription factor expression

For each drug treatment, HDFs were seeded on a 96-well plate and grown for 24 h. The cells were transduced using each lentiviral supernatant for 48 h before treatment with NecB. Cells were then treated with NecB at the indicated concentrations for 10 days. Fluorescence was evaluated every 12 h using a multiple plate reader (TriStar^2^ S LB 942, Berthold technologies, Bad Wildbad, Germany), excited at 480 nm and detected at 510 nm. The light emission was normalized by that determined in blank non-treated group and the transcription factor activity was evaluated. The regulatory networks of transcription factor activation were schematized using BTNET (http://ibtnet.korea.ac.kr/), as described previously [66].

### Statistical analysis

All measurements were performed in triplicate and values from at least three independent experiments were expressed as mean ± standard deviation. Statistical significance was assessed by analysis of variance (ANOVA) followed by Tukey’s test using GraphPad Prism Software (San Diego, CA, USA). In this study, P values ≤ 0.05 were considered statistically significant.
